# AMP-activated protein kinase controls liposaccharide-induced hyperpermeability

**DOI:** 10.1186/cc10624

**Published:** 2012-03-20

**Authors:** D Castanares-Zapatero, M Overtus, D Communi, M Horckmans, L Bertrand, C Oury, C Lecut, P Laterre, S De man, C Sommereyns, S Horman, C Beauloye

**Affiliations:** 1 Université catholique de Louvain, Cliniques universitaires Saint Luc, Brussels, Belgium; 2 Université catholique de Louvain, Institut de Recherche Expérimentale et Clinique, Brussels, Belgium; 3 Université libre de Bruxelles, Institut de Recherche Interdisciplinaire en Biologie humaine et moléculaire, Brussels, Belgium; 4 Université de Liège, Groupe Interdisciplinaire de Génoprotéomique Appliquée, Liège, Belgium

## Introduction

Organ dysfunction determines the severity of sepsis and is correlated to mortality. Endothelial increased permeability contributes to the development of organ failure. AMP-activated protein kinase (AMPK) has been shown to modulate cytoskeleton and could mediate endothelial permeability. Our hypothesis is that AMPK controls sepsis-induced hyperpermeability in the heart and is involved in septic cardiomyopathy.

## Methods

Sepsis was induced by intraperitoneal injection of liposaccharide, 10 mg/kg (LPS). Alpha-1 AMPK knockout mice (α1KO) were compared with wild-type. Vascular permeability was characterized by Evans blue extravasation. Inflammatory cytokine mRNA expression was determined by qPCR analysis. Left ventricular mass was assessed by echocardiography. In addition, to emphasize the beneficial role of AMPK on heart vascular permeability, AMPK activator (acadesine) was administered to C57Bl6 mice before LPS injection. The ANOVA test with Bonferroni's *post hoc *test and the log-rank test were used. *P *< 0.05 was considered as significant.

## Results

Increased cardiac vascular permeability was observed in the LPS group in comparison to untreated animals (2.5% vs. 16%; *P *< 0.05). The α1KO mice exhibited an increase vascular permeability after LPS injection in comparison to wild-type mice (41.5% vs. 16%; *P *< 0.05). α1KO animals had a significant mortality increase after LPS injection (70% vs. 10%; *P *< 0.05). LPS markedly induced the production of proinflammatory cytokines (TNFα, IL-1β, IL-6) that were significantly higher in the α1KO animals. More importantly, LPS treatment leads to an increased left ventricular mass in the α1KO mice within 24 hours, suggesting the onset of edema. Finally LPS-induced vascular hyperpermeability was greatly reduced after AMPK activation by acadesine (13.2% vs. 40%; *P *< 0.05).

## Conclusion

AMPK importantly regulates cardiac vascular permeability and could control the sepsis-induced cardiomyopathy. AMPK could represent a new pharmacological target of sepsis.
